# Broadband full-color monolithic InGaN light-emitting diodes by self-assembled InGaN quantum dots

**DOI:** 10.1038/srep35217

**Published:** 2016-10-13

**Authors:** Hongjian Li, Panpan Li, Junjie Kang, Jiianfeng Ding, Jun Ma, Yiyun Zhang, Xiaoyan Yi, Guohong Wang

**Affiliations:** 1 Semiconductor Lighting R&D Center, Institute of Semiconductors, Chinese Academy of Sciences, Beijing 100083, China; 2 State Key Laboratory on Integrated Optoelectronics & Optoelectronic System Laboratory, Institute of Semiconductors, Chinese Academy of Sciences, Beijing 100083, China

## Abstract

We have presented broadband full-color monolithic InGaN light-emitting diodes (LEDs) by self-assembled InGaN quantum dots (QDs) using metal organic chemical vapor deposition (MOCVD). The electroluminescence spectra of the InGaN QDs LEDs are extremely broad span from 410 nm to 720 nm with a line-width of 164 nm, covering entire visible wavelength range. A color temperature of 3370 K and a color rendering index of 69.3 have been achieved. Temperature-dependent photoluminescence measurements reveal a strong carriers localization effect of the InGaN QDs layer by obvious blue-shift of emission peak from 50 K to 300 K. The broadband luminescence spectrum is believed to be attributed to the injected carriers captured by the different localized states of InGaN QDs with various sizes, shapes and indium compositions, leading to a full visible color emission. The successful realization of our broadband InGaN QDs LEDs provide a convenient and practical method for the fabrication of GaN-based monolithic full-color LEDs in wafer scale.

InGaN-based light emitting diodes (LEDs) have been attractive due to the emission spectra covering from near-ultraviolet (NUV) to green and widely applied display technology, back lighting, and general illumination areas^1^,^2^,^3^,^4^,^5^. The most conventional method to achieve white LEDs is to combine phosphor wavelength converter with GaN LED chips, such as blue LEDs with yellow phosphor or NUV InGaN LEDs with blue/green/red phosphors^3^. Nevertheless, these methods have several disadvantages such as stokes shift energy loss, relatively short life-time, and long-term reliability of the phosphors^2^. To solve these problems, the concept of monolithic full-color InGaN LEDs without covering any phosphors has been proposed^6^,^7^,^8^,^9^,^10^. Nguyen *et al.* successfully demonstrated the fabrication of full-color LED emitting in GaN-based a dot-in-a-wire nano-structure on Si(111)^6^,^7^. Min *et al.* reported visible-color LEDs achieved by using InGaN/GaN multiple quantum wells (MQWs) formed on GaN nanostructures^8^. Shon *et al.* showed the full-color InGaN-based LEDs on amorphous substrates by pulsed sputtering^9^. Lee *et al.* presented full-color InGaN-based LEDs from non-planar InGaN/GaN MQWs grown on GaN template with truncated hexagonal pyramids^10^. Nevertheless, those full-color LEDs fabricated on multiple micro-facets or nano-strutures involve quite complicated materials growth conditions and tricky devices fabrication process, which are difficult for widely application. Other issues such as reliability and current leakage also need to be solved.

For the generation of GaN-based full-color white LEDs, the critical challenge lies on the realization of yellow or red emission. However, the luminescence efficiency of InGaN LEDs drops dramatically at this range, which is well known as “green gap”^11^,^12^,^13^. To circumvent this problem inherent in InGaN MQWs, an alternative InGaN quantum dots (QDs) structure as light-emitters has been suggested in the green or even longer spectral ranges^14^,^15^,^16^,^17^,^18^,^19^,^20^,^21^,^22^,^23^. The self-assembled QDs have significantly reduced built-in piezoelectric polarization field, leading to an alleviation of quantum confinement stark effect (QCSE) as compared to the planar QWs^14^,^15^,^16^,^17^,^18^. Furthermore, the quasi-three dimensional confinement of carriers in the InGaN/GaN QDs can reduce the rate of non-radiative recombination of carriers caused by dislocations and related defects^20^. In particular, the dependences of emission energy on QDs’ sizes and compositions provide alternative approach to tune the emission wavelength^11^,^17^,^18^,^19^,^24^. Consequently, InGaN QDs structure shows potential for the realization of broadband GaN-based LEDs.

In our study, self-assembled InGaN QDs structure is employed to achieve broadband full-color GaN-based LEDs without introducing extra processes. Full-color InGaN QDs LEDs have been successfully realized, which show superior emission light with ultra-broad spectrum span from the NUV to red range.

## Results

**Figure 1:**
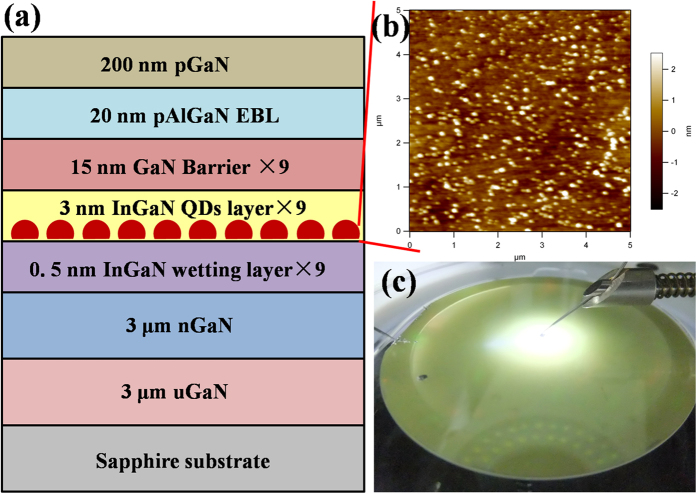
(**a**) Schematic epitaxial structure; (**b**) AMF image of surface morphology of as-grown uncapped QDs sample by 5 × 5 μm^2^ and (**c**) Image of the epitaxy wafer with a white emission by probing with forward bias.

The schematic epitaxial structure is depicted in Fig. 1(a). A 0.5-nm thin In_0.06_Ga_0.94_N wetting layer was inserted before the InGaN well layer. And a relatively high pressure of 600 mbar was employed for the following growth of MQWs growth. Under a high reactor pressure, more organic molecules will be cracked then deposited on the surface, leading to the a shorter mean free path for Indium adatoms on the top of the wetting layer, which will promotes the segregation of In adatoms for forming In-rich regions clusters in favor of 3D growth^23^. The surface morphology of uncapped QDs sample is investigated by atomic force microscope (AFM) as presented in Fig. 1(b), which indicates the feature of as-grown QDs structure with a density of ~10^10^ cm^−2^ and a diameter of 70 nm. Additionally, the image of the epitaxy wafer is captured as shown in Fig. 1(c), which emits a white light by probing with forward bias. The yellow color surface of the LEDs wafer indicates a strong indium composition within the active region. The average indium content is estimated to be about 39% by high-resolution X-ray diffraction (HRXRD) ω − 2θ measurements.

**Figure 2:**
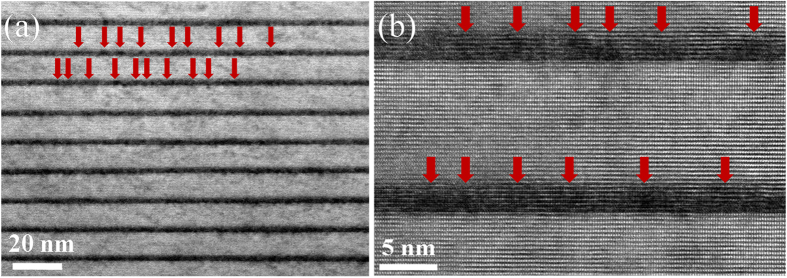
(**a**) HRTEM image of the active region and (**b**) Enlarged image of the QWs.

High resolution transmission electron microscopy (HRTEM) has been performed to investigate the detail of active region, as presented in Fig. 2(a,b). The alternately stacked bright and dark layers correspond to the GaN barriers and InGaN QWs, respectively. Distinct small QDs embedded in the well layers can be observed from Fig. 2(a) (Some of them are marked by arrows). These InGaN QDs are non-uniformly distributed with random variation in sizes and shapes, which is similar to the other reports about the ensemble properties of QDs^17^,^18^,^21^,^25^. Enlarged HRTEM image of the QWs is shown in Fig. 2(b). The average size of the QDs is estimated to be around 3 nm, which is much smaller than the AFM results. This could be caused by the migration and evaporation of indium atoms during the post-growth of the GaN barriers at a relatively higher temperature. Another possible reason can be attributed to the limited tip radius of our AFM system.

**Figure 3:**
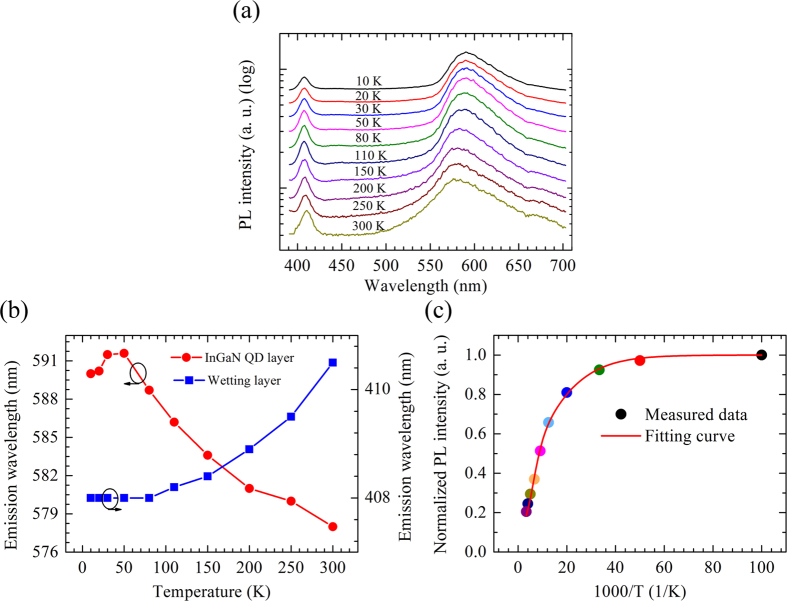
(**a**) TDPL of the InGaN QDs LEDs; (**b**) Relationship between peak emission wavelengths and temperature for the wetting layer and the InGaN QDs layer; (**c**) Normalized integrated PL intensity as a function of temperature for InGaN QDs layer.

The optical properties of the InGaN QDs LEDs are investigated using temperature-dependent photoluminescence (TDPL) measurements. Figure 3(a) describes the TDPL spectra from 10 K to 300 K. It is found that all PL spectra show a high-energy peak and a low-energy peak, which originates from the wetting layer and InGaN QDs layer, respectively. This result indicates that the InGaN QDs were formed with the Stranski-Krastanow (SK) growth mode^16^,^17^,^18^,^19^. At 10 K, the strong low-energy peak is located at 590.0 nm with a broad FWHM of 62.4 nm, whereas the week high-energy emission is peaked at 407.9 nm with a narrow line-width of 13.4 nm. It is found that there is band-tail emission at long wavelength side for the low-energy peak. Moreover, the peak emission wavelengths as a function of temperature for both wetting layer and InGaN QDs layer are plotted in Fig. 3(b). The low-energy peak presents a red-shift from 10 K to 50 K and an obvious blue-shift from 591.5 nm to 578.1 nm with the temperature increasing up to 300 K. In contrast, the high-energy peak for the wetting layer only demonstrates a slight red-shift from 407.9 nm to 410.9 nm. It is well known that the blue-shift of the emission peak during TDPL measurements has been considered as a strong evidence of carrier localization effect^25^,^26^,^27^,^28^,^29^,^30^,^31^. At a very low temperature, the excitons are less likely to move, and they are able to overcome the potential fluctuations and relax into status with lower potential minima with increasing temperature, leading to a red-shift of the emission peak. As temperature increases, the excitons escapes out of potential minima, which is known as delocalization effect and results in a blue-shift^26^,^27^,^28^. Therefore, the blue-shift of the emission peak for the InGaN QDs layer from 50 K to 300 K reveal an obvious carrier localization effect for the InGaN QDs layer. No further red-shift is observed for the InGaN QDs layer at high temperature, which is relevant to the deeper localization potential in the QDs array^31^.

The internal quantum efficiency (IQE) of the InGaN QDs white LEDs has been further investigated. Normalized integrated PL intensity of the InGaN QDs layer as a function of the reciprocal of temperature is plotted in Fig. 3(c). The IQE is calculated to be 20.6%, assuming that the nonradiative centers are completely frozen at 10 K^25^,^26^. In spite of the strong QCSE and the large dislocations densities for the high In content InGaN layer, an IQE of 20.6% is considered to be higher than the commonly reported values for InGaN-based LEDs in long wavelength range, which is related to the strong confinement effect of the carriers in QDs^6^,^16^,^32^.

The experimental data of normalized PL integrated intensity versus temperature can be fitted by the following formula to estimate the activation energy of non-radiative recombination^16^,^26^.


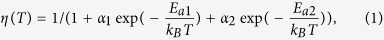


where *E*_a1_ and *E*_a2_ are the activation energies of two corresponding non-radiative recombination centers, α_1_ and α_2_ are the process rate parameters related to probability, and *k*_B_ is Boltzmann’s constant. The fitting curve is presented in Fig. 3(c), with a high squared correlation coefficient of 0.99. Active region energy *E*_a1_ and *E*_a2_ with a value of 7 meV and 68 meV can be obtained.

**Figure 4:**
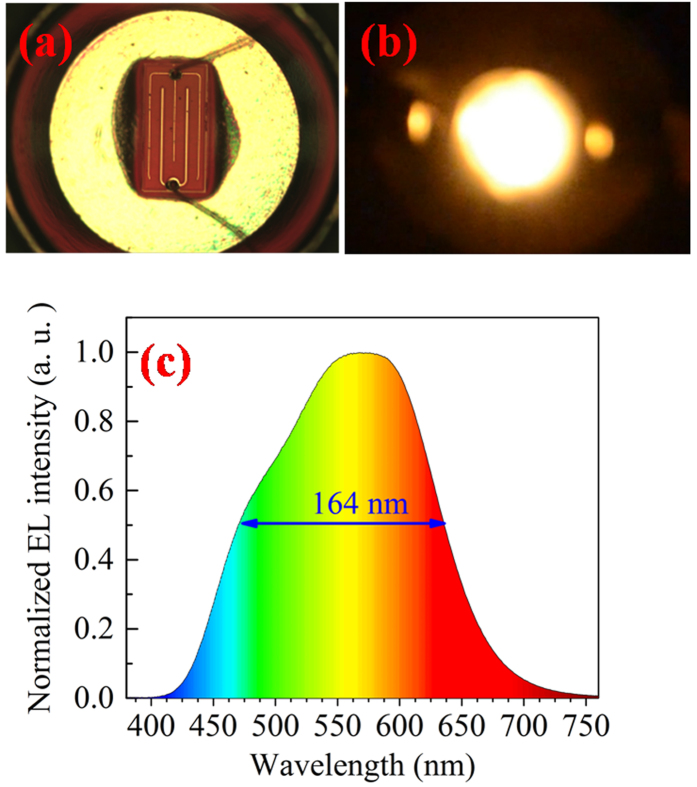
(**a**) A bare LED chip without phosphor covered; (**b**) Luminescence image of InGaN QDs LED at 200 mA and (**c**) EL spectrum with a broad line-width of 164 nm.

Furthermore, a bare chip device without phosphor covered and the electroluminescence (EL) image at 200 mA are shown in Fig. 4(a,b), respectively. A warm white emission can be clearly observed, accompanied by a color temperature of 3370 K and a color rendering index of 69.3. The EL spectrum at 200 mA is plotted in Fig. 4(c). It is worth to point out that the line-width is as large as 164 nm, and the emission spectrum is extremely broad spanned from 410 nm to 720 nm, covering the entire visible wavelength range.

**Figure 5:**
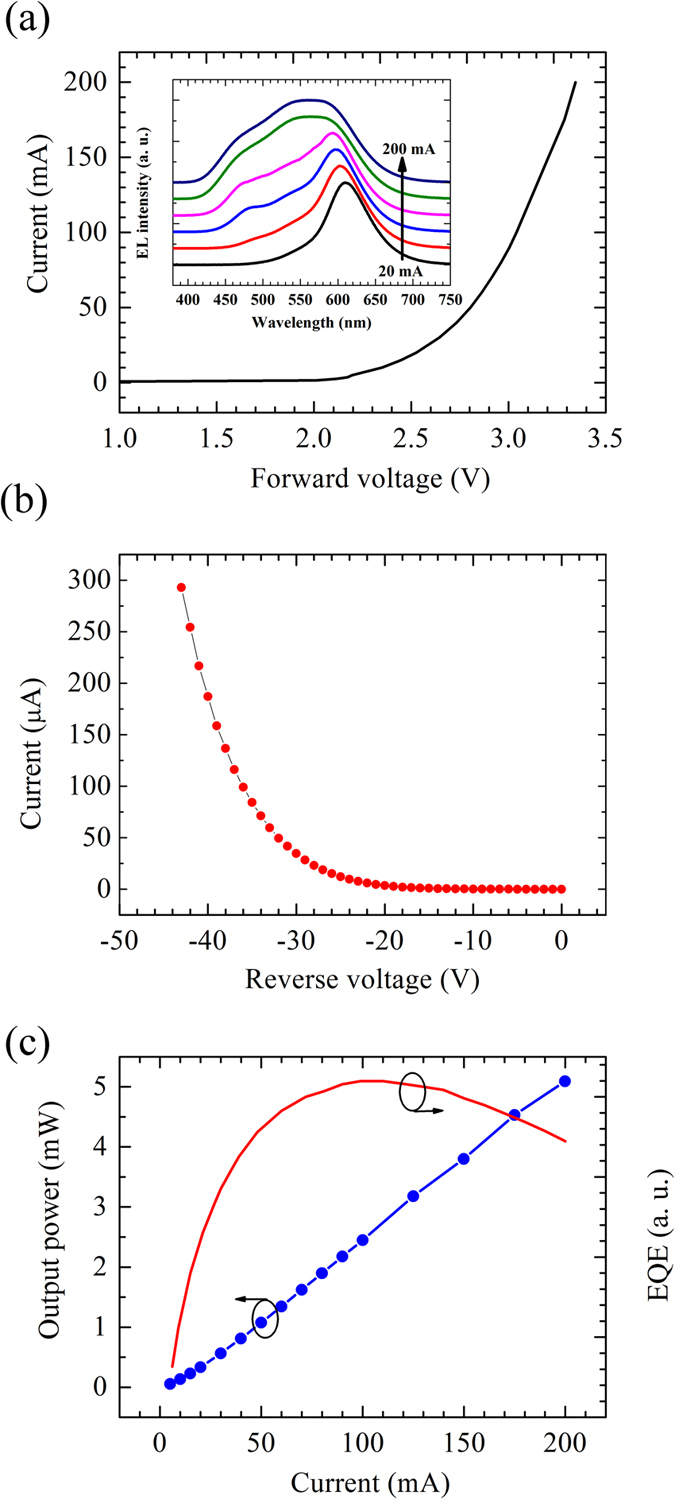
(**a**) Measured current-voltage (I–V) characteristics of InGaN QDs LEDs. The inset is excitation dependent EL spectra from 20 mA to 200 mA. (**b**) Reverse bias characteristic at a voltage −50 V to 0 V. (**c**) Characteristics of output power-current and the corresponding EQE in arbitrary units versus current.

Figure 5(a) depicts the current-voltage characteristics of the InGaN QDs LEDs. A turn-on voltage of 2.18 V is measured and the series resistance is calculated to be as low as 3.0 Ω. In particular, the leakage current is nearly zero (~50 nA) at a reverse bias voltage from −20 V to 0 V, which suggests a good *p*-*n* junction characteristic. The excitation dependent EL spectra from 20 mA to 200 mA are shown in the inset of Fig. 5(a). Single peak at long emission wavelength appears at first and the emission spectrum broadens towards high-energy side with increasing current. Finally, a broadband emission spectrum covering full visible color range has been achieved. Moreover, the characteristics of output power-current and the corresponding EQE in arbitrary units versus current are plotted in Fig. 5(b). The LEDs present an output power of 5.1 W at 200 mA and a relatively low efficiency droop, which agrees well with the previous reports about the droop effect of InGaN QDs LEDs^6^,^7^,^20^,^32^,^33^. Moreover, the repeatability and stability of our QDs based LEDs are good due to the direct growth of the wafer in MOCVD reactor and the standard devices fabrication process.

## Discussion

**Figure 6:**
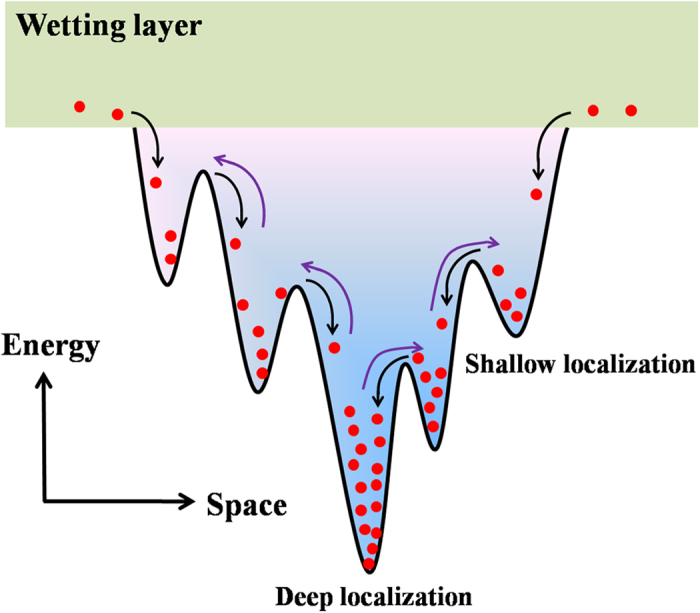
Schematic carrier recombination model in our InGaN QDs LEDs.

The broadband full-color emission of our InGaN QDs LEDs is believed to be attributed to the injected carriers captured by the localized centers of these various self-assembled InGaN QDs. Since the InGaN QDs in our experiment are ensembles of dots which vary in sizes, shapes, and compositions as shown by our TEM results, it gives rise to the ultra-broadband emission light covered by blue, green, yellow and red components by filling the different localized states. A schematic carrier recombination model is proposed, as demonstrated in Fig. 6. The deep localization centers and shallow localized centers represent the InGaN QDs with low-energy and high-energy states. The injection carriers are priority to be captured by the deep localized centers, resulting in a strong red emission. With increasing current, some of carriers start to fill the shallow energy states via hopping or delocalization, leading to the green and blue emission. Finally, a luminescence spectrum span from NUV to red range forms by the mix of emission from different localized centers^34^. It should be noted that no recombination occurs in the wetting layer in our InGaN QDs LEDs, due to the absence of localization effect as confirmed by our TDPL measurements results.

In conclusion, we have developed broadband full-color monolithic InGaN LEDs by self-assembled InGaN QDs, which emit a superior light span from NUV to red range. Especially, it is important to notice that the successful realization of our full-color InGaN QDs LEDs are in thin film wafer-scale, which suggests a more promising technology due to its convenient fabrication process, as compared to the previous reports about the full-color GaN-based LEDs on multiple micro-facets or nano-structures in the literatures.

## Methods

Growth of the InGaN QDs LEDs. We used Aixtron horizontal metal organic chemical vapor deposition (MOCVD) reactor to grow the broadband monolithic InGaN LEDs on *c*-plane (0001) sapphire substrates. The precursors were trimethylgallium (TMGa), triethylgallium (TEGa), trimethylindium (TMIn), and ammonia (NH_3_). Silane (SiH_4_) and bis-cyclopentadienyl magnesium (Cp_2_Mg) were used as n-type and p-type dopants, respectively. Before the deposition of a GaN nucleation layer, the sapphire wafer was thermally cleaned at 1150 °С under an H_2_ atmosphere for 10 min. Then a 30-nm-thick GaN buffer layer was deposited at 500 °С under a reactor pressure of 650 mbar, followed by the deposition of a 3-μm-thick undoped GaN (uGaN) layer 3-μm-thick nGaN layer (Si-doped 8 × 10^18^ cm^−3^) at 1030 °С and a reactor pressure of 300 mbar. InGaN/GaN MQWs with 9 pairs of 3 nm InGaN QDs well layers and 15 nm GaN barriers were grown at a temperature of 735 °С and 815 °С, respectively. A thin 0.5-nm In_0.06_Ga_0.94_N wetting layer was inserted before the InGaN well layer grown at the same temperature of wells. During the MQWs growth, the V/III ratio was setting at 1.15 × 10^4^, the reactor was 600 mbar and the TMIn flow for the wetting layer and the InGaN QDs layer was 8 μmol/min and 68 μmol/min, respectively. Then the temperature was increased to 920 °С for the growth of 20-nm pAlGaN electron blocking layer (EBL) and 200-nm pGaN layer (p-doping 3 × 10^19^ cm^−3^).

LEDs devices fabrication. LED devices with 0.3 mm^2^ size were fabricated using a conventional mesa structure. Indium Tin Oxide (ITO) was firstly deposited on top of pGaN as current-spreading layer. An nGaN layer was exposed by inductively coupled plasma (ICP) etching, and Cr/Pt/Au metals were deposited as p/n contact electrodes.

Characterizations. AFM measurements were performed using a Nanoscope Dimension^TM^ 3100 scanning probe microscope system. The cross-sectional of the active region were analyzed by HRTEM (Tecnai G2 F20 FEI-TEM microscope). The temperature dependence of the PL spectra was determined by cooling each sample in a closed-loop He cryostat to 10 K and then gradually heating to 300 K. A He-Cd laser 325 nm was performed, with an excitation laser power density of 210 W/cm^2^.

## Additional Information

**How to cite this article**: Li, H. *et al.* Broadband full-color monolithic InGaN light-emitting diodes by self-assembled InGaN quantum dots. *Sci. Rep.* **6**, 35217; doi: 10.1038/srep35217 (2016).
